# Editorial: Epidemiology of epilepsy and seizures

**DOI:** 10.3389/fepid.2023.1273163

**Published:** 2023-08-30

**Authors:** Zhibin Chen, Martin J. Brodie, Ding Ding, Patrick Kwan

**Affiliations:** ^1^ Department of Neuroscience, Central Clinical School, Monash University, Melbourne, VIC, Australia; ^2^ Department of Medicine—Royal Melbourne Hospital, University of Melbourne, Parkville, Melbourne, VIC, Australia; ^3^ Department of Medicine and Clinical Pharmacology, University of Glasgow, Glasgow, United Kingdom; ^4^ Department of Biostatistics and Epidemiology, Institute of Neurology, Fudan University Huashan Hospital, Shanghai, China; ^5^ Department of Neurology, Alfred Health, Melbourne, VIC, Australia; ^6^ Department of Neurology, The First Affiliated Hospital of Chongqing Medical University, Chongqing Key Laboratory of Neurology, Chongqing, China

**Keywords:** epilepsy, seizures, epidemiology, treatment gap, SUDEP, status epilepticus (SE), infantile spasm (IS)

**Editorial on the Research Topic** Epidemiology of epilepsy and seizures

Epilepsy is a chronic neurological disorder characterised by recurrent unprovoked seizures (1). It affects an estimated 50 million people worldwide with no socio-demographic boundary (2). Previous studies demonstrated the point prevalence of epilepsy to be between 4 and 10 per 1,000 persons, making it one of the most prevalent neurological conditions (2–8). The incidence rate of epilepsy is estimated around 50–60 per 100,000 person-years (2, 5), and up to 8% of people having at least one seizure in their lifetime (9). Compared to general population, people with recurrent seizures have increased physical and psychiatric comorbidities, healthcare utilisation, and excess mortality (10, 11). They have reduced quality of life (QoL) (12), and lower employment and productivity (13). Epileptic seizures account for 1% of hospital admissions and 3% of Emergency Department (ED) visits (14). The annual direct costs per patient with epilepsy is more than US$11,000, and indirect costs are more than US$3,000 (15, 16). Epilepsy is a heterogeneous with varied presentations. However, the research into the epidemiology of seizure and epilepsy is relatively limited. This Research Topic aims to collate the latest original and review articles concerning the epidemiology of epilepsy and seizures in different populations, to gain a better understanding of the prognosis of epilepsy, and to narrow the knowledge gap in long-term morbidity and mortality associated with epilepsy.

Antiseizure medications (ASMs) are the primary treatment option for epilepsy and can render up to two-thirds of treated patients seizure-free (17, 18). However, approximately 30% of newly diagnosed epilepsy patients will not attain seizure freedom while taking pharmacological treatment and may have drug-resistant epilepsy (DRE) (19), which is defined as failure to achieve seizure freedom after trials of 2 appropriate and tolerated ASM regimens, whether as monotherapy or in combination (20). In these patients, surgical interventions, such as resective surgery, may be considered. Other non-pharmacological approaches, including ketogenic diet, vagus nerve stimulation (VNS), and deep brain stimulation (DBS), have also shown efficacy as adjunctive therapies.

Treatment gap in epilepsy has long been highlighted as a public health concern. Studies have shown that approximately 80% of people with epilepsy live in low- and middle-income countries, where the treatment gap contributes substantially to the global burden of epilepsy. Up to 90% of epilepsy in these areas is untreated or undertreated, due to factors such as limited healthcare resources, cultural beliefs, social stigma, and preferential use of traditional treatments (21–23). This treatment gap is associated with poor health outcomes, life threatening conditions such as status epilepticus, and subsequently higher healthcare utilisation and healthcare costs. Recent studies also found that even in high-income countries, around a third of people with newly diagnosed epilepsy were not immediately treated (24, 25). Pellinen reviews the current literature on treatment gaps in epilepsy, highlights the issues of seizure recognition, and discusses the various barriers to timely epilepsy diagnosis and treatment in both low- and middle-income countries and high-income countries.

People with epilepsy have increased mortality attributable to both seizure-related and unrelated causes (26, 27). It is estimated around 125,000 people worldwide succumb to epilepsy each year (28). The standardised mortality rates are 1.6–3.0 times higher than the general population in high-income countries (26), and up to 7.2 times higher in low- and middle-income countries (27). Uncontrolled seizures are known to be associated with increased mortality (29). Sudden unexpected death in epilepsy (SUDEP) and status epilepticus are the most important epilepsy-related causes of mortality (30). Trinka et al. provide a comprehensive review on the mortality and life expectancy in people with epilepsy and status epilepticus. They discuss the issues in translating the widely used standardised mortality ratios into life expectancy from a methodological perspective and review the trend of mortality in epilepsy and status epilepticus. Contrary to past common beliefs that the mortality of epilepsy did not change over time, recent studies have demonstrated a decrease trend over the past half-century (31–35). This might be attributed to the early recognition of DRE and the introduction of successful non-pharmacological treatments, such as epilepsy surgery. A similar decreasing trend was found in the mortality of status epilepticus (35), which is likely benefiting from the increased application of early and aggressive treatment (36). Trinka et al. also emphasise the research gap in mortality of epilepsy and status epilepticus between high-income countries and low- and middle-income countries. Future efforts should focus on improving accessibility to specialised comprehensive epilepsy care and improving seizure control in hope to reduce premature mortality in people with epilepsy.

The incidence of epilepsy varies across different age groups and has a U-shaped bimodal distribution with the highest rates observed in young children and older adults (37). In children, the incidence is typically highest during the first year of life, and then decreases gradually. Acute seizures and infantile spasms are common in children with epilepsy and require additional care. Cui et al. investigated the knowledge, attitudes, and practices regarding the out-of-hospital management of acute seizures for children with epilepsy in Chinese families. While the families generally have a positive attitude toward the management of out-of-hospital acute seizures, they lack practical experience and related knowledge. The study underscores the urgent need for locally tailored emergency medicine, auxiliary infrastructures, and relevant resources for acute seizures in children with epilepsy. On the other hand, Liao et al. retrospectively assessed the long-term prognosis of ketogenic diet therapy in combination with ASMs for infantile spasms in a resource-limited region that has poor access to the recommended first-line hormones (corticotropin or prednisolone/prednisone) and vigabatrin therapies. While the majority of the patients have achieved seizure freedom on ketogenic diet combined with alternative ASMs, there was a significant increase in the proportion of patients who suffered from neurodevelopmental delay. However, both seizure control and neurodevelopmental outcomes were found to be similar to the standard treatment. This study sheds light on the efficacy of ketogenic diet as an adjunct with alternative ASMs for infantile spasms, warranting future clinical trials to evaluate its effectiveness as a first-line treatment.

Epilepsy and seizures affecting millions of people worldwide and impose a large health and economics burden on our community. Understanding the epidemiology of epilepsy is crucial for identifying at-risk populations, implementing preventive measures, and improving the overall management of the condition. The articles in this Research Topic present novel perspectives on epilepsy. They unveil unexpected treatment gaps in high-income countries and barriers to timely epilepsy diagnosis and treatment. Additionally, the Topic highlights decreasing trends and research gaps in the mortality of epilepsy and status epilepticus. The research also introduces insights into the positive attitudes but lack of practical experience and related knowledge among relatives for the out-of-hospital management of acute seizures in children. Moreover, it explores the effectiveness of ketogenic diet therapy for infantile spasms, offering potential avenues for improved care and treatment strategies. By addressing the risk factors, associated comorbidities, and socioeconomic implications, healthcare professionals and policymakers can work towards reducing the burden of epilepsy and providing better support to people living with epilepsy and to their families.
